# The Self, Agency and Spatial Externalizations of Inner Verbal Thoughts, and Auditory Verbal Hallucinations

**DOI:** 10.3389/fpsyt.2019.00668

**Published:** 2019-09-19

**Authors:** Massoud Stephane

**Affiliations:** Department of Psychiatry and Behavioral Sciences, Indiana University Purdue University Indianapolis, IU Health Neuroscience Center, Indianapolis, IN, United States

**Keywords:** hallucinations, psychosis, inner speech, the self, cognition

## Abstract

**Aim:** Auditory Verbal Hallucinations (AVH) are experienced as the “voices” of others (O-AVH) or self (S-AVH) in internal space/inside the head (IS-AVH) or external space (ES-AVH), and are considered to result from agency and spatial externalizations of inner speech. Both types of externalizations are conflated, and the relationship between these externalizations and AVH experiences is unclear. In this paper, I investigate the relationship between cognitive agency and spatial externalizations and between these externalizations and the types of AVH experience.

**Method:** Twenty-five patients with history of AVH and 24 matched healthy controls performed agency and spatial distinction tasks: distinction between self-generated (read) (S) sentences and other-generated (O) sentences, and between sentences read silently (experienced in internal space, IS) and sentences read aloud (experienced in external space, ES). Regression analyses between misattribution biases (S-O vs. IS-ES, and O-S vs. ES-IS) were obtained. t tests were used to compare misattribution biases between AVH subtypes (S-AVH vs. O-AVH, and IS-AVH vs. ES-AVH).

**Results:** Regressions suggest that agency distinction is independent from spatial distinction in both groups. O-AVH and S-AVH subgroups differed only with respect to S-O bias, and IS-AVH and ES-AVH subgroups differed only with respect to IS-ES bias.

**Conclusion:** These results suggest that agency and spatial externalizations of inner speech are independent at phenomenological and cognitive and levels; and that these externalizations are co-related across levels. I discuss the implications of these findings in the wider context of research on AVH and on the experience of self.

## Introduction

Auditory Verbal Hallucinations (AVH), i.e. auditory perceptions of speech without corresponding external object (1) are symptoms of many psychiatric and medical illnesses (2). AVH are also encountered in over 5% of the general (non-clinical) population (3), and often alienate affected individuals; those who seek treatment generally find partial relief with current therapeutics (4).

While the mechanisms of AVH remain elusive, research consistently implicated disordered language processes (5) and, in particular, disorders of generation of inner speech (inner verbal thoughts) (6). In health, inner speech is recognized as one’s own and experienced in internal space (inside the head). Although not invariably, subjects with AVH report hearing the “voices” of others and often experience these “voices” in external space—hereafter, phenomenological agency and spatial externalizations, respectively (7, 8). From the perspective of inner speech generation disorder, these aspects of AVH experiences point to agency and spatial externalizations of inner verbal thoughts at a cognitive level. Consistently, neuropsychiatric research has shown evidence for both types of externalizations (9, 10).

Cognitive agency externalization was extensively investigated using source memory tasks to examine the distinction between self-related (S) and other-related (O) stimuli—S/O distinction. Different populations of hallucinating subjects were studied with various experimental designs and various types of stimuli, and the results were somewhat inconsistent. For example, some studies examined the distinction between self- and other-generated motor actions (non-speech stimuli) (11) while others examined the distinction between speech pre-recorded in one’s own voice and that pre-recorded in the voice of other (speech perception design) (12). Furthermore, with different experiments, studies examined the distinction between speech generated during the experiment by self or other (speech generation designs) (13–18). While the results of these studies were inconsistent, meta-analysis of studies up to 2012 suggests S–O misattribution in hallucinating subjects (9). However, if indeed AVH result from speech generation disorder, non-speech stimuli and speech perception experiments are sub-optimal for the evaluation of S/O distinction of speech relevant to AVH. Using a speech generation paradigm and addressing a number of limitations in prior studies, we have previously shown significant S–O, but not O–S, misattribution in patients with AVH (19). S–O misattribution, in our study, was not related to general distinction ability, confounds (such as medication doses), or illness severity; which suggests that cognitive inner speech agency externalization (S–O misattribution) is a trait deficit for AVH.

Cognitive spatial externalization received less attention. Research has examined the distinction between speech experienced in internal space (IS) and that experienced in external space (ES)—IS/ES distinction—in AVH; and, just as in S/O distinction experiments, speech perception and speech generation paradigms were used. In the former, subjects distinguished between speech delivered *via* headphones simulating IS perception and that simulating ES perception (20). In the latter, subjects distinguished between sentences they silently read (experienced in IS) and sentences they read aloud (experienced in ES) (10, 21, 22). All speech generation studies (10, 21, 22), unlike that of speech perception (20), reported IS/ES distinction impairment. Differences in the populations studied [schizophrenia patients (10, 21, 22) vs. healthy subjects prone to all types of hallucinations but not necessarily AVH (20)] might also contribute to the discrepancy. Furthermore, as argued above, speech generation paradigms might provide more accurate appraisal of speech disorders pertinent to AVH. In a study, we used a speech generation experiment and found both IS–ES and ES–IS misattributions that were not related to general recognition capacity, potential confounds, or clinical severity scores (10). These results suggest a trait deficit affecting the processes of the spatial localization of inner speech percepts in patients with AVH.

The above considerations suggest that AVH phenomenological agency and spatial externalities (hearing the “voices” of others in external space, respectively) result from cognitive agency and spatial externalizations of inner speech (S–O and IS–ES misattributions, respectively). However, just as other-attributed “voices” have been generally considered synonymous to “voices” experienced in external space, S/O and IS/ES misattributions have been conflated (20). Such understanding is inconsistent with historical conceptualization (23) and more recent evidence (24) of multiplicity of the domains of the experience of self. More importantly, it is inconsistent with phenomenological evidence indicating that AVH agency and spatial externalities are not ubiquitous or synonymous.

Research indicates that AVH are phenomenologically heterogeneous, and could be experienced as the “voices” of self (S-AVH) or other (O-AVH), in internal space (IS-AVH) or external space (ES-AVH) (7, 8, 25). Furthermore, using multidimensional scaling (MDS) (26), we have previously constructed a map of the phenomenological space of AVH based on co-occurrences between AVH phenomenological variables, and shown that agency (S or O) and spatial (IS or ES) experiences of AVH are independent dimensions (8). In other words, IS-AVH are not necessarily experienced as S-AVH; and, similarly, ES-AVH are not necessarily experienced as O-AVH. We hypothesized that these independent dimensions reflect independent underlying neural dysfunctions; and the “where” and “what” dual auditory pathways were subsequently suggested as candidates (27). It was also suggested that the above dimensionality reflects independent underlying cognitive deficits: subjective origin (self or non-self) and subjective source (inner space or outer space) (28). To date, there are no experimental evidence to evaluate these phenomenological implications. In the present study, I investigate the relationship between S/O agency distinction and IS/ES spatial distinction; and between the phenomenological and cognitive levels of agency and spatial externalizations.

## Methods

### Human Subjects

Twenty-five patients (24 males and 1 female) schizophrenia/schizoaffective patients with history of AVH, and 24 (23 males, 1 female) healthy control subjects were included in this study. Patients were recruited at the psychiatry clinic at the VA Medical Center (Minneapolis, MN), and controls were recruited through flyers posted at the VA. The experimental protocol was approved by the Institutional Review Boards at both the VA Medical Center and the University of Minnesota; and subjects gave written informed consent before participation in this research. Patients and controls received a diagnostic assessment using the Structured Clinical Interview for DSM IV (SCID) (29) as well as an assessment of premorbid intellectual functioning using the National Adult Reading Test (NART) (30). Hallucinations were evaluated using the computerized binary Scale of Auditory Speech Hallucinations (cbSASH) (31) to identify phenomenological subtypes of AVH based on the S, O, IS, and ES experiences of the “voices.” Patients were also evaluated with the Brief Psychiatric Rating Scale (BPRS) (32), the Scale for the Assessment of Negative Symptoms (SANS) (33), and the Scale for the Assessment of Positive Symptoms (SAPS) (34). Furthermore, the illness durations were obtained from records reviews, and chlorpromazine equivalent doses of antipsychotic medications were computed (35). Subjects had short training sessions right before data collection to familiarize themselves with the experimental tasks described below.

### Experimental Tasks

Agency (S/O) distinction: the task was carried out in 6 blocks of about 3 min duration each. Each block consisted of two sequential phases for presentation and testing. In the presentation phase, subjects alternately read aloud sentences that appeared on the computer screen for 3,500 ms or listened to sentences pre-recorded in a neutral tone in the voice of another while the screen remained blank. The gender of the voice of the heard sentences matched that of the subject. Five read and five heard sentences were presented in random order in each block. In the testing phase, these ten sentences were mixed with five new sentences and visually presented on the computer screen one at a time in random order. Subjects were instructed to determine the source (agency) of the sentence: read = self-generated (S), heard = other-generated (O), or new = no agency coding (NC). The test sentences remained on the screen until responses were made. This task previously demonstrated significant S–O misattributions in schizophrenia patients (19).

**Figure 1:**
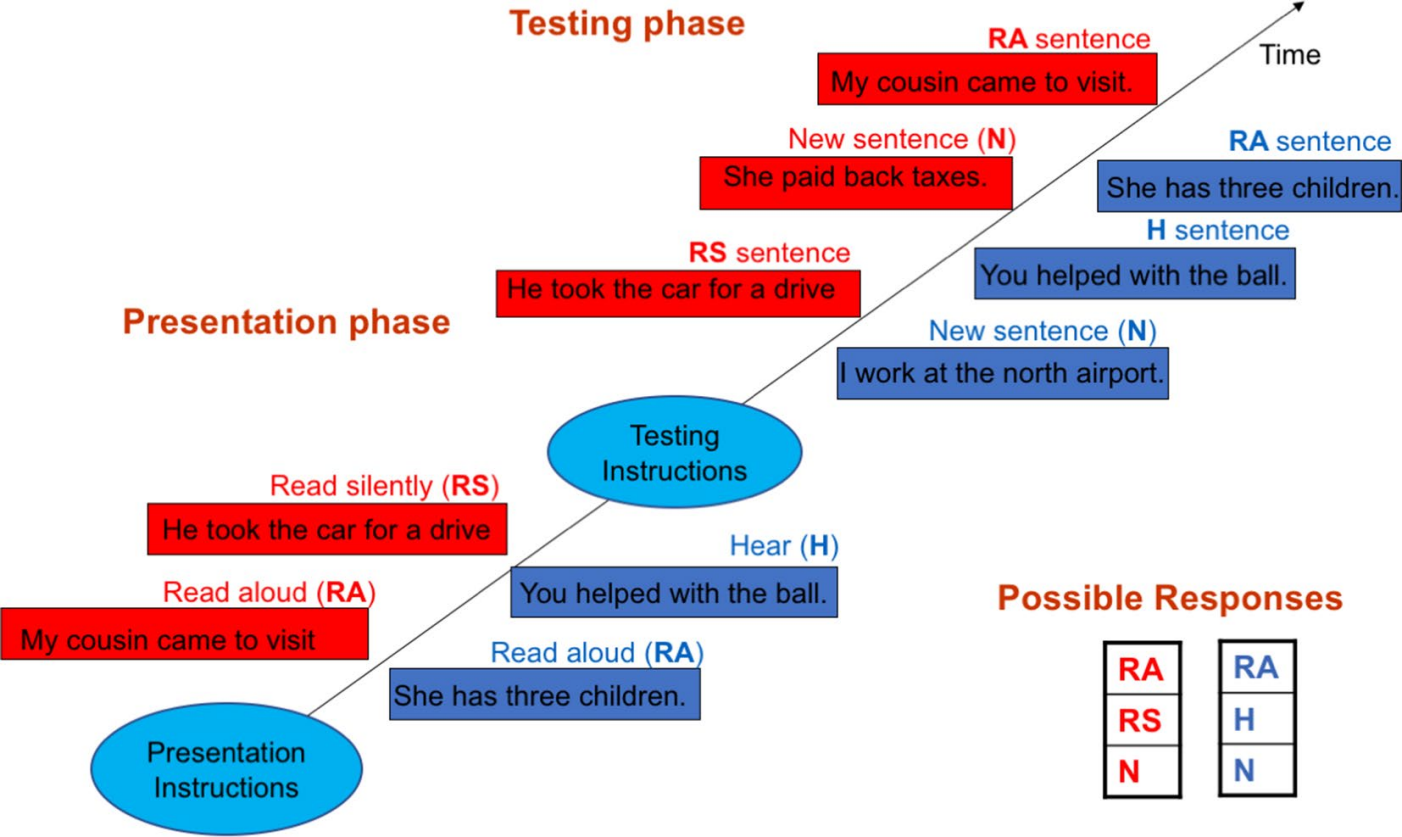
Outlines of the self/other (S/O)—blue rectangles—and internal space/external space (IS/ES) distinction tasks—red rectangles. In the S/O task during the presentation phase, subjects alternately read aloud (RA) sentences appearing on the screen and heard (H) pre-recorded sentences played back while the screen remained blank. In the testing phase, the RA and H sentences were visually presented along with new (N) sentences one a time in random orders. Subjects were instructed to identify through button press the sentence type: RA, H or N. The IS/ES task followed the same general design. However, in the presentation phase, subjects instead alternately read aloud (RA) and read silently (RS) sentences visually presented on the screen. In the testing phase, the RA and RS sentences were visually presented along with new sentences (N), and subjects were instructed to identify the sentence type: RA, RS or N.

Spatial (IS/ES) distinction: The experiment was also carried out in 6 blocks, and each block consisted of sequential presentation and test phases. The presentation phase consisted of two parts. In one part, subjects read aloud five sentences appearing on the computer screen one at a time. In the other part, subjects read silently five sentences similarly presented. Each sentence remained on the screen for 3,500 ms, and the two parts were presented in random orders across blocks. During testing, these ten sentences were mixed with five new sentences and visually presented one at a time in a random order. Subjects were instructed to distinguish between the three types of sentences: read silently = experienced in IS, read aloud = experienced in ES, or new = not spatially coded (NC). The test sentences remained on the screen until responses were made. This task previously demonstrated both IS–ES and ES–IS misattributions in schizophrenia patients (10). **Figure 1** outlines both tasks.

The sentences were chosen from magazines in the patient waiting room in the clinic. On average, the sentences were five-words long, had neutral affective content, and belonged to general categories such as sports and daily living. They were written in the first-, second-, and third-person with equal probability. Both procedures were programmed using E-prime (Psychology Software Tool, Pittsburgh, PA) coding for correct and actual responses, which allows for computation of response accuracy as well as error types.

Both tasks are based on a standard psycholinguistic procedure (reading) to evaluate speech (36). While social speech is different from inner speech, reading is a practical laboratory procedure to evaluate inner speech as both inner speech and social speech share a common developmental precursor (37, 38) and, to some extent, common neural resources (6). In particular, reading in the experimental design described above allows the investigation aspects of verbal thoughts implicated in AVH—agency and spatial experiences. Furthermore, as both tasks were similarly designed, both are likely to call upon similar cognitive resources other than those involving S/O and IS/ES distinctions. Accordingly, the relationships between S/O and IS/ES distinction operations could be examined by comparing task performances.

## Analyses

All analyses were carried out with SPSS Version 24 (IBM SPSS; Armonk, New York) and included the following:

### Characteristics of the Experimental Samples

Independent samples t tests were used to examine group differences with respect to potential confounding factors including age, personal and parental levels of education and premorbid intellectual functioning.

### Group Differences in Agency and Spatial Distinction Capacities

t tests were used to examine the group differences in the percent of correct recognition of sentences that were agency (S or O) coded—agency distinction capacity—and spatially (IS or ES) coded—spatial distinction capacity. t tests were also used to examine group differences in the percent of correct recognition of sentences that were NOT agency or spatially coded (NC), which reflects general recognition capacity independently from agency and spatial distinction capacity.

### The Relationship Between Agency and Spatial Distinction Capacities

These analyses were carried out with two methods. In the first method, error scores for the following types of misattribution errors were first computed: S–O (read sentences recognized as heard), O–S (heard sentences recognized as read), IS–ES (sentences read silently recognized as read aloud), ES–IS (sentences read aloud recognized as read silently). Subsequently, linear regression analyses were carried out between S–O errors and IS–ES errors, and between O–S errors and ES–IS errors, with S–O and O–S errors as the dependent variables and IS–ES and ES–IS as the explanatory variables, respectively. Group interaction term was included in both regressions. Given the similarity of experimental designs of the S/O and IS/ES distinction tasks, it is likely that, other than S/O and IS/ES distinctions, both tasks call upon largely similar memory and cognitive capacities. Therefore, a lack of relationship between these errors would point to the independence of the operations of S/O and IS/ES distinction, rather than to differences in memory and/or cognitive capacities.

With the second method, we considered the possibility that the two tasks differ in cognitive and memory requirements, in which case error scores comparisons would not accurately reflect the comparison of the operations of S/O and IS/ES distinction. To address this possibility, we obtained measures of S–O, O–S, IS–ES and ES–IS misattribution bias that are independent of memory and cognitive capacities. For this purpose, we computed the ratio of the misattribution errors of interest to all other type of errors in each task. For example:

S–O misattribution bias = S–O errors/S–O errors + S–N errors + O–S errors + O–N errors + N–S errors + N–O errors.

Where S–O, S–O are as defined above, and S–N = sentences read recognized as new, O–N = sentences heard recognized as new, and N–S, N–O errors = new sentences recognized as read or heard, respectively. Whereas S–O and O–S errors depends on both agency distinction failures as well as memory failures; S–N, O–N, and N–S, N–O errors only reflect memory failures. Accordingly, the ratio provides specific measure of externalizations and internalizations biases (agency externalization in the above example). Subsequently, linear regression analyses were carried out between S–O and IS–ES misattribution biases and between O–S and ES–IS misattribution biases, as in the first method.

### The Relationships Between Agency and Spatial Experiences of AVH

The following subgroups of patients were first defined based on the agency (S or O) and spatial (IS or ES) experiences of AVH as evaluated by the cbSASH: S-AVH, O-AVH, IS-AVH and ES-AVH. The cbSASH showed that AVH were experienced as the “voices” of Self (2 patients), Self or Other alternately (6 patients) and Other (16 patients). As those who experience AVH as either their own voice or that of others are likely to have a degree of integrity of the self-agency, the first two types of AVH experiences were combined in the S-AVH subgroup. With respect to phenomenological spatial externalization, the cbSASH showed that AVH were experienced in IS (9 patients), in ES (5 patients) and alternately in IS or ES (7 patients). Three patients were not sure about the spatial location of their “voices.” As those with alternate IS and ES experience of AVH are likely to have a degree of phenomenological spatial externalizations, the latter two types of spatial experience of AVH were combined in the ES-AVH. Subsequently, the relationship between phenomenological agency and spatial experiences was examined using chi square test.

### The Relationships Between the Phenomenological and Cognitive Levels of Agency and Spatial Externalizations

Independent samples t tests were used to compare the S-AVH and O-AVH subgroups with respect the theoretically related measures of cognitive agency distinction failure (S–O and O–S misattribution biases) and with respect to theoretically unrelated cognitive spatial distinction failures (IS–ES and ES–IS misattribution biases) as control conditions. Similarly, IS-AVH and ES-AVH subgroups were compared with respect to cognitive spatial distinction failures (condition of interest) and cognitive agency distinction failures (control condition.)

## Results

### Characteristics of the Samples

**Table 1** summarizes the demographic variables of the two groups. There were no significant differences in age, personal level of education, or mean parental level of education. However, the two groups differed in their performance on the NART (patients, 100.2 ± 8.3; controls, 108.5 ± 7.3, p < 0.001). In the patient group, the mean scores on the BPRS, SANS, and SAPS were (44 ± 10), (8 ± 4), and (8.7 ± 4.5), respectively. The mean duration of illness was (23 ± 12) years, and the mean chlorpromazine equivalent dose of medication was (314 ± 187) mg.

**Table 1:** Mean and standard deviation of age, parental level of education (Parents Ed), national adult reading test full score (NART-FS) and personal level of education (Ed) in patients and controls.

Group		Age	Parents Ed	NART-FS	Ed
Patients	Mean	52.7	11.5	100.2	13.7
	SD	11	3.5	8.3	2.3
Controls	Mean	52.7	11.4	108.5	14.5
	SD	11.9	2.8	7.3	2.4

### Group Differences in Agency and Spatial Distinction Capacities

Patients significantly differed from controls in the percent of correct recognition of agency (S/O) coded sentences p < 0.02 and the percent of recognition of spatially coded sentences p < 0.03, multiple comparisons corrected (39). The percent of correct recognition of sentences that were not agency or spatially coded did not differentiate groups p > 0.74. These results are consistent with our previous work (10, 19) where these questions were comprehensively investigated, and suggest specific impairment in agency and spatial distinction capacities in AVH. **Table 2** outlines the findings.

**Table 2:** Group differences in the percent of correct recognition of sentences that were agency (S/O) or spatially (IS-ES) coded and those that were not agency or spatially coded (NC).

	Coding	Mean	Mean	Std. Error	Sig.
		pt	ctl	Dif.	
**S/O distinction**	S/O coded	71.2	83.2	4	.004
	NC	92.4	94.2	2.6	.5
**IS/ES distinction**	IS-ES coded	59.3	69.3	4.2	.02
	NC	96	96.2	2	.9

The mean of percent of accurate responses in patients (pt) was significantly lower from that of controls (ctl) for agency and spatially coded sentences (bold font) but not for sentences that were not agency or spatially coded.

### The Relationship Between Agency and Spatial Distinction Capacities

**Figure 2:**
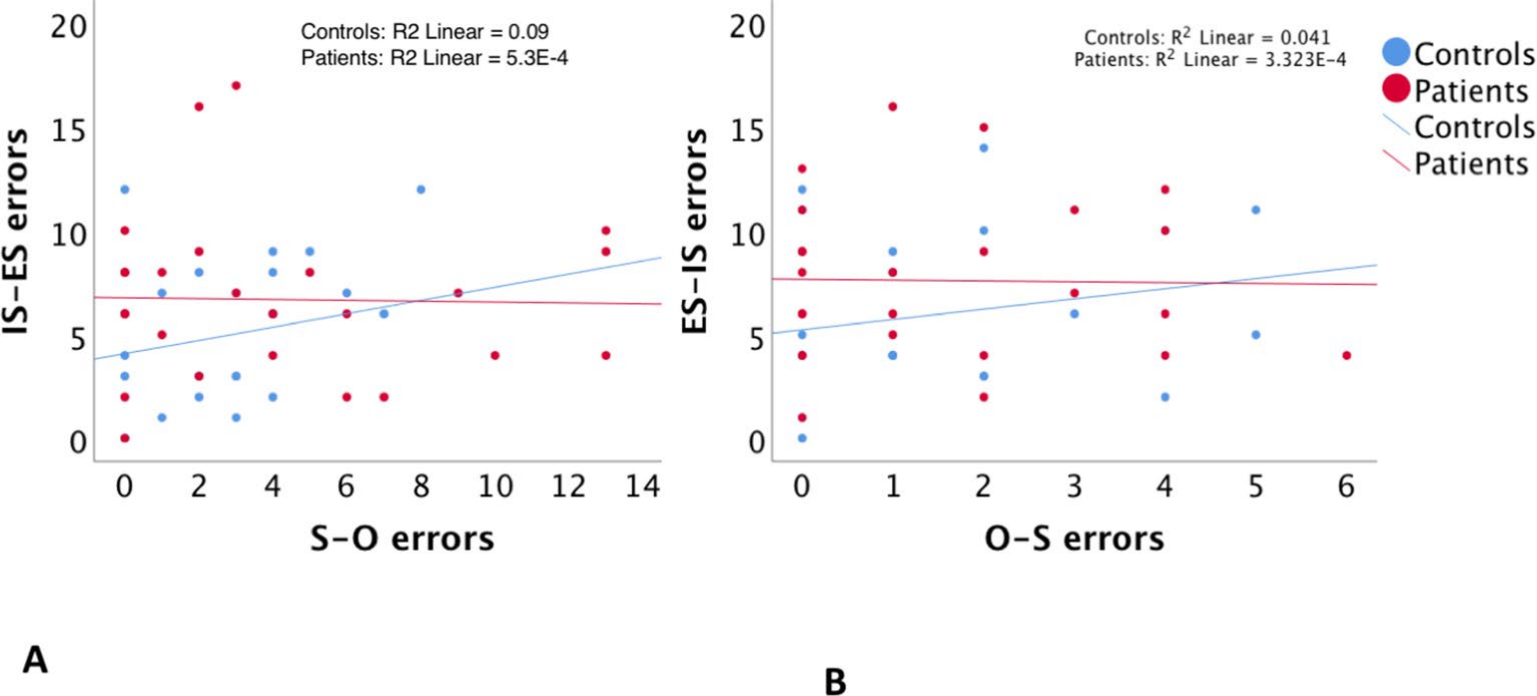
The relationship between self–other misattribution errors (S–O errors) and internal space–external space misattribution errors (IS–ES errors) **(A)**, and between other–self misattribution errors (O–S errors) and external space–internal space misattribution errors (ES–IS errors) **(B)**.

As expected, regression analyses using error scores showed that the null hypothesis of independent agency externalization (O–S errors) and spatial externalization (IS–ES errors) could not be rejected (P > 0.9). Similarly, there was no relationship between agency internalization (O–S errors) and spatial internalization (ES–IS errors) (p > 0.8). Group interactions were negative in both regressions at a P > 0.9 and P > 0.8, respectively (**Table 3** **A**, **Figure 2**) Accordingly, additional regression analyses to account for potential confounding factors related to group differences (the NART, BPRS, SANS, and SAPS scores, and the estimates of duration of illness and of chlorpromazine equivalent doses of medications) were not carried out.

**Table 3:** Results of linear regression analyses using misattribution error scores **(A)** and misattribution bias scores **(B)**.

A	Unstandardized coefficients		Standardized coefficients		
	Beta	Std. Error	Beta	t	p
*S–O errors vs. IS–ES errors*	0.02	0.21	0.02	0.11	0.92
*Group X S–O errors vs. IS–ES errors*	0.00	0.17	0.00	0.02	0.98
*O–S errors vs. ES–IS errors*	0.02	0.08	0.05	0.26	0.80
*Group X O–S errors vs. ES–IS errors*	−0.01	0.06	−0.03	−0.18	0.86
B					
	Beta	Std0. Error	Beta	t	p
*S–O Bias vs. IS/ES Bias*	−0.14	0.25	−0.09	−0.57	0.57
*Group X S–O Bias vs. IS–ES Bias*	0.17	0.21	0.13	0.79	0.44
*O–S Bias vs. ES–IS Bias*	−0.05	0.08	−0.09	−0.57	0.55
*Group X O–S Bias vs. ES–IS Bias*	0.08	0.08	0.16	10.1	0.29

S, Self; O, Other; IS, internal space; ES, external space.

**Figure 3:**
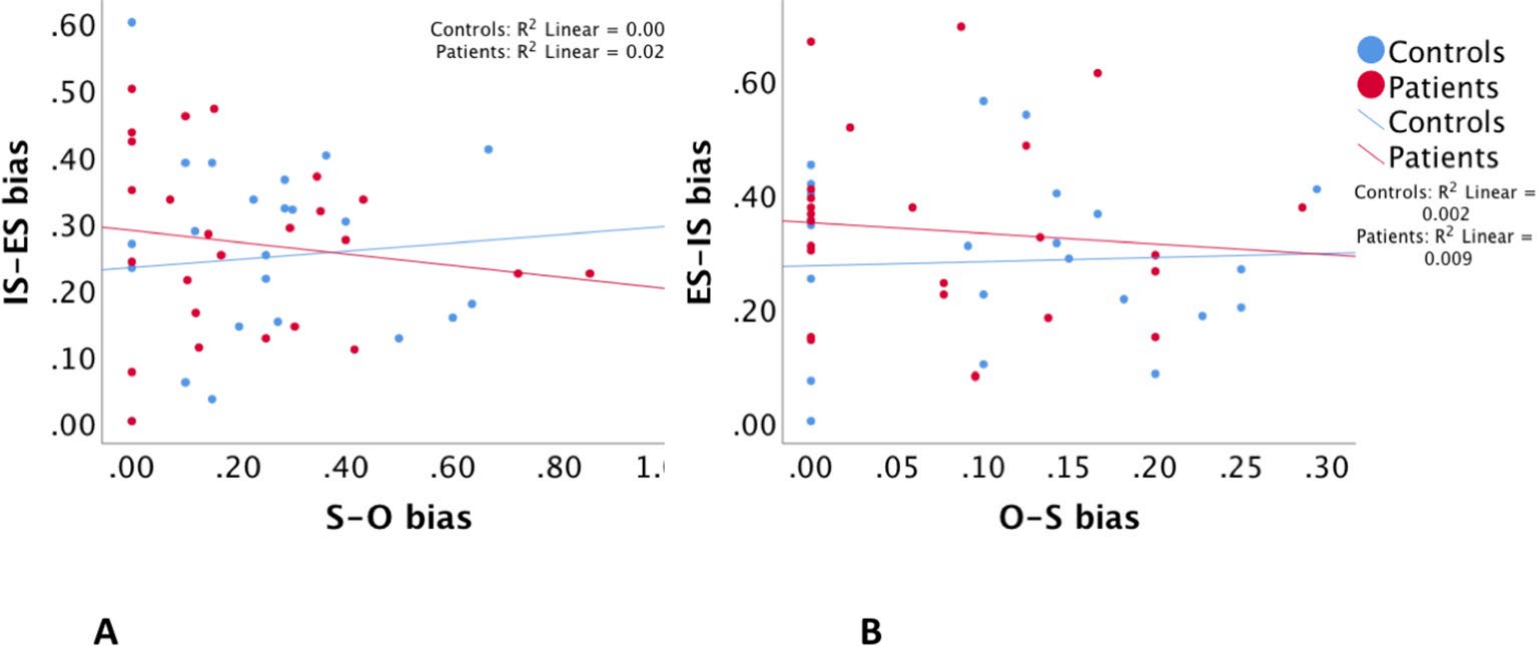
The relationship between self–other misattribution bias (S–O bias) and internal space–external space misattribution bias (IS–ES bias) **(A)**, and between other–self misattribution bias (O–S bias) and external space–internal space misattribution bias (ES–IS bias) **(B)**.

Regression analyses with estimates of misattribution biases were consistent with those with misattribution errors described above. Regressions between S–O bias and IS–ES bias and between O–S bias and ES–IS bias were both insignificant with a p > 0.57 and p > 0.55, respectively. Group interaction effects were also not significant in both regressions with a p > 0.4 and p > 0.3, respectively. Accordingly potential group-related confounding factors were not tested. **Table 3** **B** and **Figure 3** illustrate these findings.

### The Relationships Between Agency and Spatial Experiences of AVH

Consistent with our previous work (8), chi square between agency (S-AVH, O-AVH) and spatial (IS-AVH, ES-AVH) experiences of AVH was insignificant (1.180, df 1, P > 0.28) suggesting the independence of these experiences of AVH. Interestingly, S-AVH were more than twice as frequent to be experienced in ES than in IS (**Table 4**).

**Table 4:** Associations between agency and spatial experiences of AVH. Chi square did not rule of the null hypothesis of no associations at a p > 0.28. Also note S-AVH are more likely to be experienced in external space.

		Spatial experience		
		ES-AVH	IS-AVH	Total
**Agency experience**	O-AVH	7	8	15
	S-AVH	5	2	7
	Total	12	10	22

### The Relationship Between the Phenomenological and Cognitive Levels of Externalizations

**Figure 4:**
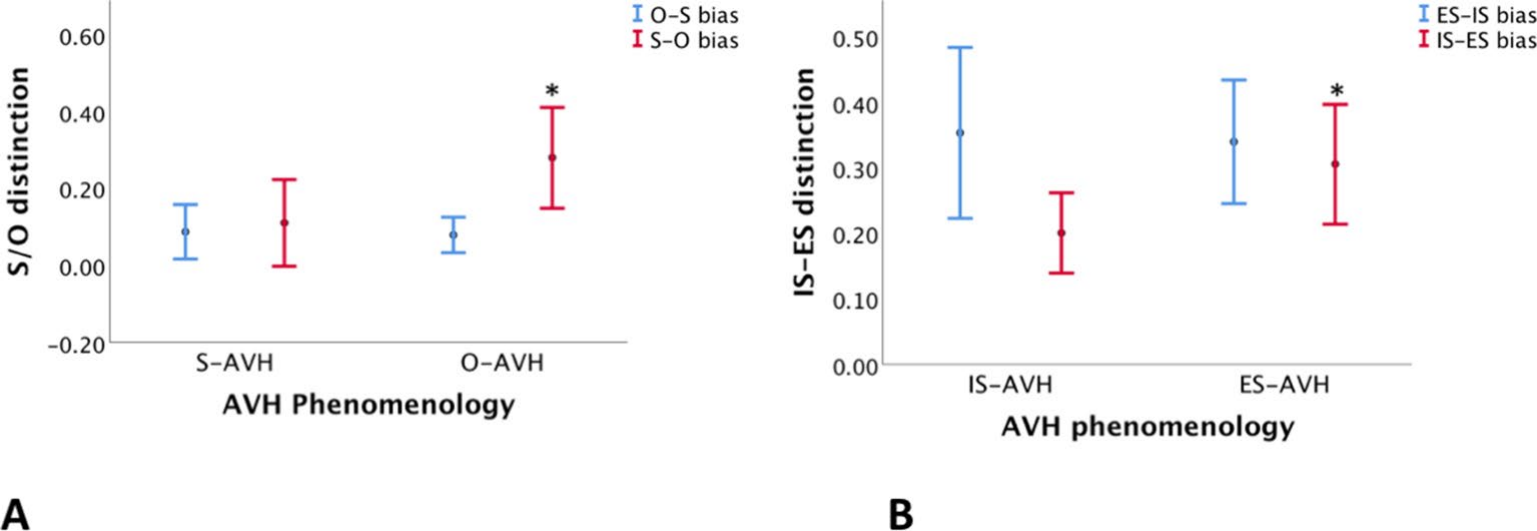
**(A)** Differences in self–other (S–O)—red lines—and other–self (O–S)—blue lines—misattribution bias between subgroups of patients defined according to the agency experience of AVH: “voices” experienced as those of Self (S-AVH), and “voices” experienced as those of Other (O-AVH)). Only S–O bias difference was significant. **(B)** Differences in internal space–external space (IS–ES)—red lines—and external space–internal space (ES–IS)—blue lines—misattribution bias between subgroups of patients defined according to the spatial experience of AVH: “voices” experienced in internal space (IS-AVH), and “voices” experienced in external space (ES-AVH)). Only IS–ES bias was *significant*. *denote significance.

Patients with phenomenological agency externalization (O-AVH) showed significantly higher S–O, but not O–S, misattribution bias than those without phenomenological agency externalization (S-AVH) p < 0.04 (**Table 5**, **Figure 4A**) Furthermore, control variables (IS–ES and ES–IS misattribution biases) were not significantly different.

**Table 5:** Comparison of S–O and O–S misattribution biases between subgroups of patients defined according to AVH phenomenology: O-AVH, S-AVH (upper panel). Comparison of IS–ES and ES–IS misattribution biases between subgroups of patients defined according to AVH phenomenology: IS-AVH, ES-AVH (upper panel).

–	AVH experience	N	Mean	Std. deviation	Std. error mean
S–O misattribution bias*	O-AVH	16	**0.28**	0.25	0.06
	S-AVH	8	**0.11**	0.13	0.05
O–S misattribution bias	O-AVH	16	0.08	0.09	0.02
	S-AVH	8	0.09	0.08	0.03
IS–ES misattribution bias*	ES-AVH	12	**0.30**	0.14	0.04
	IS-AVH	11	**0.20**	0.09	0.02
ES–IS misattribution bias	ES-AVH	12	0.35	0.20	0.05
	IS-AVH	11	0.34	0.15	0.04

S, Self; O, Other; IS, internal space; ES, external space. *and bold font indicate significance.

Similarly, patient with phenomenological spatial externalization (ES-AVH) showed significantly higher IS–ES, but not ES–IS, misattribution bias than those without phenomenological spatial externalization (IS-AVH) p < 0.05 (**Table 5**, **Figure 4B**); and control variables (S–O and O–S misattribution biases) were not significant. Interestingly, mean S–O misattribution bias was 0.31 in the IS-AVH subgroup and 0.13 in the ES-AVH subgroup reaching a trend for significance p < 0.08. This trend is consistent with the independence of agency and spatial processes.

## Discussion

The present study suggests that in health and disease, agency and spatial experiences of inner speech are related to independent operations; and, as such, these operations independently fail in disease—patients with AVH—resulting in agency and spatial externalizations of inner verbal thoughts, respectively.

At a cognitive level in both groups, agency externalization (S–O bias) was highly independent from spatial externalization (IS–ES bias) and the same relationship was found between agency and spatial internalizations (O–S and ES–IS biases, respectively). Furthermore, consistent with our previous work (8), the agency and spatial experiences of AVH were independent from one another. Moreover, the phenomenological and cognitive levels of agency and spatial externalizations were co-related. S–O bias, but not O–S bias, was significantly higher in patients with O-AVH relative to patients with S-AVH; and, similarly, IS–ES bias, but not ES–IS bias, was significantly higher in patients with ES-AVH relative to patients with IS-AVH. Of further importance, spatial distinction failures (IS–ES and ES–IS biases) did not differentiate patient subgroups defined based on agency experiences of AVH (S-AVH and O-AVH); and, similarly, agency distinction failures (S–O and O–S biases) did not differentiate patient subgroups defined based on spatial experiences of AVH (IS-AVH and ES-AVH). This is a further indication that cognitive agency and spatial externalizations are directly related to the respective agency and special experiences of AVH, rather than ubiquitous deficits.

Multiple neural deficits as abnormal inner speech agency due to a corollary discharge deficit (11), heightened top down information processing (40) and impaired selective attention and context memory (41, 42) have been reported in subjects with AVH. However, it is generally accepted that any given deficit fails short of accounting for the wide range of the phenomenological diversity of AVH experiences (43). For example, Hunter (44) accurately argued that abnormal inner speech agency does not explain ES experience of inner speech. Previously, it was suggested that subject-specific combinatoric associations of multiple neural deficits could explain AVH experiences variable across subjects (28, 45); and the present data support this theory. Specifically, the presence/absence of cognitive agency externalization, cognitive spatial externalization, or both determines the types of hallucinatory experience: hearing the “voices” of others in IS, hearing the “voices” of others in ES, hearing one’s own “voice” in IS or hearing one’s “voice” in ES. The data also show that in some patients, hallucination instances could be either S-AVH or O-AVH, and similarly alternate IS-AVH and ES-AVH instances are also noted. This likely reflects a milder degree of impairments of S/O and IS/ES distinction in these patients relative to those with exclusively O-AVH and IS-AVH experiences, respectively. Such account is suggested by current knowledge of the neural dysfunctions in schizophrenia. The literature point to abnormalities such as dysconnectivity and abnormal laterality (13, 14), which usually result in inefficient operations (here S/O and IS/ES distinction) rather than complete cessation of these operations.

To my knowledge, there is no prior similar line of inquiry or similar findings in the literature. However, the findings of this study are consistent with AVH imaging research and with the neuroscience of the experience of the self. AVH imaging has shown distinct neural bases for phenomenological and cognitive agency and spatial externalizations. In one study, the morphology of brain structures implicated in S/O distinction—the temporoparietal Junction (TPJ) and the inferior parietal lobule—were dependent on S-AVH or O-AVH subtypes (46). Whereas patient with S-AVH had a morphology similar to that of healthy subjects (Steinmetz type 1 morphology) (47), patient with O-AVH did not. In another study, it was shown that white matter volume of the right TPJ was higher in patients with IS-AVH relative to both patients with ES-AVH and healthy controls (48). Moreover, during hallucinations, IS-AVH relative to ES-AVH were associated with higher activity in the left planum temporal and right middle frontal gyrus (49). Furthermore, our group has recently investigated neural activity associated with the operations of S/O agency and IS/ES distinction. We have shown that whereas higher activity of midline structures implicated in S/O distinction was observed in other-generated speech relative to self-generated speech in controls, the inverse was the case in hallucinating patients, which suggests propensity for S/O agency tags reversal during inner speech in AVH (50). with respect to IS/ES distinction, patients relative to controls have shown higher activity in the precuneus, a component of the “where” auditory pathway that is also involved in spatial imagery, during IS speech percepts relative to both rest and ES speech percepts. This suggests that IS speech percepts could be at times processed as ES percepts in AVH (51).

A common trend in AVH research is to consider inner speech externalization as a unitary phenomenon that reflect a deficit in source-memory (also referred to as self-monitoring, or reality-monitoring). The latter, reality monitoring, appears to equate *external reality* with *reality* and to imply that *internal reality* (subjective experience) is unreal. As such, this terminology may not be an accurate description of the underlying issue in AVH—a mismatch between internal and external realities. While self or source monitoring deficit could accurately describe the underlying neural dysfunction in AVH, the present data suggest that the scope of the monitoring deficit in AVH involves more than inner speech agency, it also involves inner space experience of inner speech. Moreover, our recent imaging research showed that neural abnormalities in AVH patients was associated with the actual agency and spatial experiences of inner speech, not with the memory of these experiences (the recall of who spoke or of where the voice was coming form) (50, 51). These considerations suggest that AVH could be understood as abnormal experiences of the self.

From a Cartesian perspective, the subjective experience of the self is immune to error (52). One may be wrong about what one thinks about the world but one is never wrong about the fact that one thinks what he/she thinks; that is: *if I think an apple is green, I can be wrong about the color of the apple but I cannot be wrong about the fact that I think an apple is green*. AVH, as inner speech generation disorders, challenge this philosophical perspective in multiple ways; that is: *I thought of something but it is not me who was thinking and the thoughts seem outside of my head, let aside I am hearing, not thinking and many other aspects of hallucinations*. This discrepancy appears to stem from the difference between one’s self and one’s brain; and AVH are not the only brain disorder that violates the Cartesian perspective of the self.

In patients with non-dominant hemisphere strokes, the paralyzed hemi-body is often considered as alien (53); and in epilepsy patients (54) as well as with stimulation of the temporoparietal junction (55), out-of-body experiences are reported. In these instances, not unlike AVH, there appears to be a dissociation between the experiences of self-agency and internal space/one’s body. Although, in other symptoms of psychosis such as thought insertion (56) and passivity symptoms (57), there appears to be concomitant disorders of agency and internal space. It should be noted that the multiplicity of domains of the experience of self has been conceptualized since William James (23); and self-domain specific impairments in schizophrenia—reduced Ichheit (first-person *givenness*) and *Meinhaftigkeit* (*mineness*)—have been described by Kurt Schneider [cited by Parnas and Henriksen (58)]. Furthermore, recent neuroscience research, echoing William James, points to multiple self domains (such as minimal self, embodied self, narrative self, agency and ownership) (59–62), and to domain-specific brain activity (24, 63).

### Limitations and Suggestions for Future Research

It should be noted that this study is based on a small sample, and does not address intermediate AVH phenotypes (those with alternate S-AVH and O-AVH, and alternate IS-AVH and ES-AVH). Therefore, studies with larger number of subjects would be needed for replication and finer phenomenological subtyping of AVH. Furthermore, AVH are frequent in schizophrenia but are not limited to this illness. It is unclear whether the present findings would generalize to AVH in other psychiatric, medical, or general populations. The present study could be considered as a proof of concept that AVH phenomenology informs about the underlying cognitive, and possibly neural, impairments. As such, future cross-diagnostic and phenomenology informed AVH research could result in better understanding of AVH mechanisms as well as personalized treatment of these symptoms.

## Conclusion

The present study suggests that agency and spatial externalizations of inner verbal thoughts are not ubiquitous or synonymous in AVH. Phenomenological agency externalization (hearing “voices” of other) appears to result from cognitive agency externalization (S-O bias) and phenomenological spatial externalization (hearing “voices” outside the head) appears to result from cognitive spatial externalization (IS/ES bias). Furthermore, both types of externalization could be understood in the wider context of abnormal experiences of the self. These considerations suggest need to disambiguate these externalizations from one another in brain level research of AVH mechanisms. It also suggests that cognitive remediation targeting agency and spatial distinction impairments as well as metacognitive integration of self domains might be effective treatment for AVH.

## Data Availability

The datasets for this manuscript are not publicly available because will provide data if requested, data is stored on secure computer. Requests to access the datasets should be directed to stephmas@iupui.edu.

## Ethics Statement

The protocol conformed to ethical priniciples and was approved by Institutional Review Boards at both the VA Medical Center and the University of Minnesota.

## Author Contributions

MS is the sole author of this paper; MS has developed the concept and design of the study and supervised data acquisition by research assistants. MS also has analyzed the data, drafted the manuscript and designed the figures.

## Funding

This work was supported by funds from the VA Medical Center, the William and Martha Muska Foundation, and Indiana University Medical School.

## Conflict of Interest Statement

The author declares that the research was conducted in the absence of any commercial or financial relationships that could be construed as a potential conflict of interest.

## References

[1] Esquirol E Hallucination. In: Al A.e., editor. Dictionnaire des sciences médicales par une société de médecins et de chirurgiens, vol. 20 Panckoucke (1817).

[2] Stephane M Starkstein S Pahissa J Psychosis in general medical and neurological conditions. In: Waters F Stephane M, editors. Assessment of Psychosis: a reference and rating scales for research and practice. Routledge New York, NY (2015).

[3] van Os J Linscott RJ Myin-Germeys I Delespaul PLK A systematic review and meta-analysis of the psychosis continuum: evidence for a psychosis proneness-persistence-impairment model of psychotic disorder. Psychol Med (2009) 39(2):179–95. 10.1017/S0033291708003814 18606047

[4] Sommer IEC Slotema CW Daskalakis ZJ Derks EM Dirk Blom JD van der Gaag M The treatment of hallucinations in schizophrenia spectrum disorders. Schizophrenia Bull (2012) 38(4):704–14. 10.1093/schbul/sbs034 PMC3577047 22368234

[5] Zmigrod L Garrison JR Carr J Simons JS The neural mechanisms of hallucinations: a quantitative meta-analysis of neuroimaging studies. Neurosci Biobehav Rev (2016) 69:113–23. 10.1016/j.neubiorev.2016.05.037 27473935

[6] Stephane M Barton SN Boutros NN Auditory verbal hallucinations and dysfunction of the neural substrates of speech. Schizophrenia Res (2001) 50:63–80. 10.1016/S0920-9964(00)00150-X 11378315

[7] Nayani TH David AS The auditory hallucination: a phenomenological survey. Psychol Med (1996) 26(1):177–89. 10.1017/S003329170003381X 8643757

[8] Stephane M Thuras P Nassrallah H Georgopoulos AP The internal structure of the phenomenology of auditory verbal hallucinations. Schizophrenia Res (2003) 61:185–93. 10.1016/S0920-9964(03)00013-6 12729870

[9] Waters F Woodward T Allen P Aleman A Sommer I Self-recognition deficits in schizophrenia patients with auditory hallucinations: a meta-analysis of the literature. Schizophrenia Bull (2012) 38(4):741–50. 10.1093/schbul/sbq144 PMC3406529 21147895

[10] Stephane M Kuskowski M McClannahan K Surerus C Nelson K Evaluation of inner-outer space distinction and verbal hallucinations in schizophrenia. Cogn Neuropsychiatry (2010) 15:441–50. 10.1080/13546801003619884 20349369

[11] Frith CD Done DJ Experiences of alien control of schizophrenia reflect a disorder of central monitoring of action. Psychol Med (1989) 19:359–64. 10.1017/S003329170001240X 2762440

[12] Allen PP Johns LC Fu CH Broome MR Vythelingum GN McGuire PK Misattribution of external speech in patients with hallucinations and delusions. Schizophrenia Res (2004) 69:277–87. 10.1016/j.schres.2003.09.008 15469199

[13] Woodward TS Menon M Whitman JC Source monitoring biases and auditory hallucinations. Cogn Neuropsychiatry (2007) 12:477–94. 10.1080/13546800701307198 17978935

[14] Cahill C Silbersweig D Frith C Psychotic experiences induced in deluded patients using distorted auditory feedback. Cogn Neuropsychiatry (1996) 1:201–11. 10.1080/135468096396505 16571486

[15] Johns LC Rossell S Frith C Ahmad F Hemsley D Kuipers E Verbal self-monitoring and auditory verbal hallucinations in patients with schizophrenia. Psychol Med (2001) 31:705–15. 10.1017/S0033291701003774 11352372

[16] Johns LC Gregg L Allen P McGuire PK Impaired verbal self-monitoring in psychosis: effects of state, trait and diagnosis. Psychol Med (2006) 36:465–74. 10.1017/S0033291705006628 16403240

[17] Versmissen D Janssen I Johns LC McGuire PK Drukker M Campo J Verbal self-monitoring in psychosis: a non-replication. Psychol Med (2007) 37:569–76. 10.1017/S0033291706009780 17250778

[18] Costafreda SG Brébion G Allen P McGuire PK Fu CH Affective modulation of external misattribution bias in source monitoring in schizophrenia. Psychol Med (2008) 1:1–4. 10.1017/S0033291708003243 18377674

[19] Stephane M Kuskowski M McClannahan K Surerus C Nelson K Evaluation of speech misattribution bias in schizophrenia. Psychol Med (2010) 40(5):741–8. 10.1017/S003329170999081X 19719896

[20] McKague M McAnally KI Puccio F Bendall S Jackson HJ Hearing voices inside and outside the head: spatial source monitoring in participants prone to auditory hallucinations. Cogn Neuropsychiatry (2012) 17:506–26. 10.1080/13546805.2012.670503 22571383

[21] Harvey PD Reality monitoring in mania and schizophrenia. The association of thought disorder and performance. J Nerv Ment Dis (1985) 173:67–73. 10.1097/00005053-198502000-00001 3968548

[22] Franck N Rouby P Daprati E Dalery J Cardine MM Georgieff N Confusion between silent and overt reading in schizophrenia. Schizophrenia Res (2000) 41:357–64. 10.1016/S0920-9964(99)00067-5 10708345

[23] James W Principles of psychology. New York, NY: Henry Holt and Company (1890). 10.1037/10538-000

[24] Araujo HF Kaplan J Damasio H Damasio A Neural correlates of different self domains. Brain Behav (2015) 5(12):e00409. 10.1002/brb3.409 26807336 PMC4714646

[25] McCarthy-Jones S Trauer T Mackinnon A Sims E Thomas N Copolov DL A new phenomenological survey of auditory hallucinations: evidence for subtypes and implications for theory and practice. Schizophrenia Bull (2014) 40(1):231–5. 10.1093/schbul/sbs156 PMC3885292 23267192

[26] Kruskal JB Wish M Multidimensional scaling. Newbury Park: Sage Publications (1978). 10.4135/9781412985130

[27] Badcock JC The cognitive neuropsychology of auditory hallucinations: a parallel auditory pathways framework. Schizophrenia Bull (2010) 36(3):576–84. 10.1093/schbul/sbn128 PMC2879695 18835839

[28] Larøi F Woodward TS Hallucinations from a cognitive perspective. Harv Rev Psychiatry (2007) 15(3):109–17. 10.1080/10673220701401993 17510830

[29] First MB Spitzer RL Gibbon M Williams JBW Structured clinical interview for Axis I DSM-IV Disorders — patient edition (SCID-I/P, Version 2.0). New York: Biometrics Research Department, New York State Psychiatric Institute (1995).

[30] Blair JR Spreen O Predicting premorbid IQ: a revision of the national adult reading test. Clin Neuropsychol (1989) 3:129–36. 10.1080/13854048908403285

[31] Stephane M Pellizzer G Roberts S McClannahan K Computerized binary scale of auditory speech hallucinations (cbSASH). Schizophrenia Res (2006) 88:73–81. 10.1016/j.schres.2006.05.020 16901679

[32] Overall JE Gorham DR The brief psychiatric rating scale (BPRS). Psychol Rep (1962) 10:799–812. 10.2466/pr0.1962.10.3.799

[33] Andreasen NC Scale for the Assessment of Negative Symptoms (SANS). Iowa City: University of Iowa Press (1983).

[34] Andreasen NC Scale for the Assessment of Positive Symptoms (SAPS). Iowa City: University of Iowa Press (1984).

[35] Woods SW Chlorpromazine equivalent doses for the newer atypical antipsychotics. J Clin Psychiatry (2003) 64(6):663–7. 10.4088/JCP.v64n0607 12823080

[36] Levelt WJM Speaking From Intention to Articulation. Cambridge, MA: MIT Press (1989).

[37] Piaget J The language and thought of the child. New York: Meridian Books (1955).

[38] Vygotsky LS Mind in Society, the Development of Higher Psychological Processes. Cambridge: Harvard University Press (1978).

[39] Benjamini Y Hochberg Y Controlling the false discovery rate: A practical and powerful approach to multiple testing. J Royal Stat Soc Ser B (1995) 57:289–300. 10.1111/j.2517-6161.1995.tb02031.x

[40] Aleman A Bocker KBE Hijman R de Haan EHF Kahn RS Cognitive basis of hallucinations in schizophrenia: role of top-down information processing. Schizophrenia Res (2003) 64:175–85. 10.1016/S0920-9964(03)00060-4 14613682

[41] Waters FA Badcock JC Maybery MT The ‘who’ and ‘when’ of context memory: different patterns of association with auditory hallucinations. Schizophrenia Res (2006) 82:271–3. 10.1016/j.schres.2005.12.847 16417987

[42] Brebion GG David AS Jones HM Ohlsen R Pilowsky LS Temporal context discrimination in patients with schizophrenia: associations with auditory hallucinations and negative symptoms. Neuropsychological (2007) 45:817–23. 10.1016/j.neuropsychologia.2006.08.009 16996090

[43] Laroi F de Haan S Jones S Raballo A Auditory verbal hallucinations: dialoguing between the cognitive sciences and phenomenology. Phenomenol Cogn Sci (2010) 9(2):225–40. 10.1007/s11097-010-9156-0

[44] Hunter MD Locating voices in space: a perceptual model for auditory hallucinations? Cogn Neuropsychiatry (2004) 9:93–105. 10.1080/13546800344000174 16571576

[45] Stephane M Auditory verbal hallucinations result from combinatoric associations of multiple neural events. Front Hum Neurosci (2013) 7(329):1–8. 10.3389/fnhum.2013.00239 23755004 PMC3668292

[46] Plaze M Mangin JF Paillère-Martinot ML Artiges E Olié JP Krebs MO Who is talking to me? self-other attribution of auditory hallucinations and sulcation of the right temporoparietal junction. Schizophrenia Res (2015) 169:95–100. 10.1016/j.schres.2015.10.011 26463879

[47] Steinmetz H Ebeling U Huang YX Kahn T Sulcus topography of the parietal opercular region: an anatomic and MR study. Brain Lang (1990) 38(4):515–33. 10.1016/0093-934X(90)90135-4 2375980

[48] Plaze M Paillère-Martinot ML Penttilä J Januel D de Beaurepaire R Bellivier F Where do auditory hallucinations come from?A brain morphometry study of schizophrenia patients with inner or outer space hallucinations. Schizophrenia Bull (2011) 37(1):212–21. 10.1093/schbul/sbp081 PMC3004180 19666833

[49] Looijestijn J Diederen K Goekoop R Sommer I Daalman K Kahn R The auditory dorsal stream plays a crucial role in projecting hallucinated voices into external space. Schizophrenia Res (2013) 146:314–9. 10.1016/j.schres.2013.02.004 23453584

[50] Stephane M Burton P Meriwether D Yoon G Other tags for Self—generated speech in patients with auditory verbal hallucinations, an fMRI study. Schizophrenia Res (2018) 200:410–1. 10.1016/j.schres.2018.06.056 29970289

[51] Stephane M Burton P Meriwether D Yoon G Spatial externalization of inner verbal thoughts in auditory verbal hallucinations, an fMRI study. Schizophrenia Res (2018) 200:417–9. 10.1016/j.schres.2018.06.063 30029831

[52] Descartes R Méditations métaphysiques. Paris: Flammarion (1992).

[53] Moro V Pernigo S Tsakiris M Avesani R Edelstyn NM Jenkinson PM Motor versus body awareness: voxel-based lesion analysis in anosognosia for hemiplegia and somatoparaphrenia following right hemisphere stroke. Cortex (2016) 83:62–77. 10.1016/j.cortex.2016.07.001 27494375

[54] Greyson B Fountain NB Derr LL Broshek DK Out-of-body experiences associated with seizures. Front Hum Neurosci (2014) 8(65):1–11. 10.3389/fnhum.2014.00487 24592228 PMC3923147

[55] Blanke O Mohr C Michel CM Pascual-Leone A Brugger P Seeck M Linking out-of-body experience and self processing to mental own-body imagery at the temporoparietal junction. J Neurosci (2005) 25(3):550–7. 10.1523/JNEUROSCI.2612-04.2005 PMC6725328 15659590

[56] Sterzer P Mishara AL Voss M Heinz A Thought insertion as a self-disturbance: an integration of predictive coding and phenomenological approaches. Front Hum Neurosci (2016) 10:502. 10.3389/fnhum.2016.00502 27785123 PMC5060939

[57] Graham-Schmidt KT Martin-Iverson MT Waters FAV Self- and other-agency in people with passivity (first rank) symptoms in schizophrenia. Schizophrenia Res (2018) 192:75–81. 10.1016/j.schres.2017.04.024 28416095

[58] Parnas J Henriksen M Disturbance of the experience of self—a phenomenologically based approach. In: Waters F Stephane M, editors. The Assessment of Psychosis: A Reference Book and Rating Scales for Research and Practice. Routledge (2014).

[59] Gallagher S Philosophical conceptions of the self: implications for cognitive science. Trends Cogn Sci (2000) 4(1):14–21. 10.1016/S1364-6613(99)01417-5 10637618

[60] Damasio A Self Comes to Mind: Constructing the Conscious Brain. New York, NY: Knopf Doubleday Publishing (2010).

[61] Farrer C Frith CD Experiencing oneself vs another person as being the cause of an action: the neural correlates of the experience of agency. Neuroimage (2002) 15(3):596–603. 10.1006/nimg.2001.1009 11848702

[62] Pettersson-Yeo W Allen P Benetti S McGuire P Mechelli A Dysconnectivity in schizophrenia: Where are we now? Neurosci Biobehav Rev (2011) 35:1110–24. 10.1016/j.neubiorev.2010.11.004 21115039

[63] Stephane M Hagen MC Lee JT Uecker J Pardo PJ Kuskowski M About the mechanisms of auditory verbal hallucinations: A positron emission tomographic study. J Psychiatry Neurosci (2006) 31(6):396–405. 17136217 PMC1635803

